# Predicting Potentially Suitable Habitats and Analyzing the Distribution Patterns of the Rare and Endangered Genus *Syndiclis* Hook. f. (Lauraceae) in China

**DOI:** 10.3390/plants14152268

**Published:** 2025-07-23

**Authors:** Lang Huang, Weihao Yao, Xu Xiao, Yang Zhang, Rui Chen, Yanbing Yang, Zhi Li

**Affiliations:** 1 Guizhou Academy of Forestry, Guiyang 550005, China; huang_lng@163.com (L.H.); ywh19831027397@163.com (W.Y.); chenruiqiu@yeah.net (R.C.); yangyanbingjh@163.com (Y.Y.); 2 Key Laboratory for Biodiversity Conservation in Karst Mountain Area of Southwestern China, National Forestry and Grassland Administration, Guiyang 550005, China; 3 College of Forestry, Guizhou University, Guiyang 550005, China; xiaoxu199801@163.com; 4 Guiyang Wildlife and Plant Protection Station, Guiyang 550002, China; zhangyanggzu@163.com

**Keywords:** MaxEnt model, *Syndiclis*, habitat suitability, distribution pattern

## Abstract

Changes in habitat suitability are critical indicators of the ecological impacts of climate change. *Syndiclis* Hook. f., a rare and endangered genus endemic to montane limestone and cloud forest ecosystems in China, holds considerable ecological and economic value. However, knowledge of its current distribution and the key environmental factors influencing its habitat suitability remains limited. In this study, we employed the MaxEnt model, integrated with geographic information systems (ArcGIS), to predict the potential distribution of *Syndiclis* under current and future climate scenarios, identify dominant bioclimatic drivers, and assess temporal and spatial shifts in habitat patterns. We also analyzed spatial displacement of habitat centroids to explore potential migration pathways. The model demonstrated excellent performance (AUC = 0.988), with current suitable habitats primarily located in Hainan, Taiwan, Southeastern Yunnan, and along the Yunnan–Guangxi border. Temperature seasonality (bio7) emerged as the most important predictor (67.00%), followed by precipitation of the driest quarter (bio17, 14.90%), while soil factors played a relatively minor role. Under future climate projections, Hainan and Taiwan are expected to serve as stable climatic refugia, whereas the overall suitable habitat area is projected to decline significantly. Combined with topographic constraints, population decline, and limited dispersal ability, these changes elevate the risk of extinction for *Syndiclis* in the wild. Landscape pattern analysis revealed increased habitat fragmentation under warming conditions, with only 4.08% of suitable areas currently under effective protection. We recommend prioritizing conservation efforts in regions with habitat contraction (e.g., Guangxi and Yunnan) and stable refugia (e.g., Hainan and Taiwan). Conservation strategies should integrate targeted in situ and ex situ actions, guided by dominant environmental variables and projected migration routes, to ensure the long-term persistence of *Syndiclis* populations and support evidence-based conservation planning.

## 1. Introduction

Climate is a fundamental ecological factor shaping the natural geographic distribution of species. Its ongoing changes exert profound effects on ecosystem structure and function, community composition, and species spatial patterns, thereby posing a severe threat to global biodiversity [1,2]. Consequently, understanding biotic responses to climate change has emerged as a major focus in ecological research [3], particularly in biogeographical studies exploring the relationship between plant distribution and climatic variables [4]. The Sixth Assessment Report of the Intergovernmental Panel on Climate Change (IPCC) projects that global warming will accelerate, with surface temperatures likely to exceed 1.5 °C above pre-industrial levels by 2030 [5]. The intensifying climate crisis, along with increasing anthropogenic disturbances, has led to significant ecological disruptions, rendering many habitats unsuitable for native species [6]. As a result, numerous species are undergoing range shifts or experiencing habitat fragmentation and contraction [7,8], while some face heightened risks of local or global extinction due to limited adaptive capacity [9]. Plant species show considerable variation in ecological plasticity: widespread taxa with broader niches are generally more resilient, whereas narrowly distributed or endangered species with specialized habitat requirements tend to be more vulnerable to environmental stressors [10]. As climate regimes continue to shift, plant physiological and ecological traits may undergo significant changes, and species unable to adapt promptly are increasingly at risk of extinction [11,12]. In this context, accurately predicting the distribution patterns and suitable habitats of rare and endangered species is critical for informing conservation planning. Establishing protection measures within climatically favorable regions and monitoring temporal trends in habitat suitability can provide vital insights for designing adaptive, forward-looking conservation strategies. Nonetheless, our understanding of how rare plants respond to climate change across space and time remains limited, underscoring the urgent need for predictive approaches to support biodiversity conservation in a changing world [13].

Species distribution models (SDMs) are widely used tools in biodiversity and conservation research, offering a statistical framework to correlate species occurrences with environmental variables to assess habitat preferences and ecological tolerances [14]. By integrating species records with climatic, topographic, and edaphic variables, SDMs facilitate the identification of key environmental drivers, the inference of ecological niche characteristics, and the projection of suitable habitats under both current and future climate scenarios [14]. In recent years, SDMs have become essential in conservation planning for rare and endangered species, supporting applications such as habitat suitability mapping, conservation priority setting, and invasive species risk assessment [15,16]. Common modeling approaches include Bioclim, the Genetic Algorithm for Rule-set Prediction (GARP), Ecological Niche Factor Analysis (ENFA), Random Forest (RF), and the Maximum Entropy (MaxEnt) model [17]. Among these, MaxEnt has gained widespread use due to its high predictive performance, flexibility, and ability to perform reliably with small sample sizes and limited occurrence data [18,19]. Notably, MaxEnt is particularly effective in modeling potential distributions for narrowly distributed or data-deficient plant species [20]. For example, the suitable habitat of *Picea chihuahuana* Martínez is projected to decrease significantly under future warming scenarios, primarily influenced by minimum temperature of the coldest month, cold- and warm-season precipitation, and mean annual temperature [21]. In contrast, *Horsfieldia hainanensis* Merr. is expected to expand its suitable range under future climate conditions, with suitability mainly determined by the coldest month’s minimum temperature, annual temperature range, and the normalized difference vegetation index (NDVI) [22]. Similarly, the distribution of *Camellia luteoflora* Y. K. Li ex Hung T. Chang & F. A. Zeng is primarily affected by average temperature and precipitation during the hottest and coldest seasons [23]. Research on *Camphora* Fabr. species has shown that cold-season temperatures, temperature seasonality, and annual precipitation are key factors influencing their distribution, with suitable habitats showing varying degrees of expansion or contraction under future climate scenarios [24]. These case studies highlight the effectiveness of MaxEnt in predicting the distributional responses of threatened plants under changing climates, providing a valuable tool for biodiversity conservation.

*Syndiclis*, a genus within the Subtribe Beilschmiediineae of the Lauraceae family, currently comprises twelve recognized species, with eleven endemic to China and primarily distributed across Yunnan, Guizhou, Guangxi, Hainan, Guangdong, and Hong Kong [25]. These species are ecologically specialized, favoring humid environments and predominantly occurring in karst limestone landscapes, where natural populations tend to be highly fragmented and sparse. Characterized by low fruit set, limited seed dispersal, and poor seedling survival, *Syndiclis* populations exhibit inherent vulnerability and a trend toward decline [26]. Beyond their ecological importance, *Syndiclis* species possess considerable economic value, as their seeds are rich in high-quality oils suitable for both industrial and edible uses, and some taxa have been proposed for afforestation in karst regions [27]. Recent research has explored various facets of the genus, including morphology [28,29], phylogeny [30,31,32], biogeography [33], population ecology [26], and genomics [34], with a strong focus on taxonomic delimitation. *Syndiclis* is believed to have originated in the early Eocene (~51 Ma), with X.W. Li proposing a local origin in Southwestern China [35]. The type species, *S. paradoxa* Hook. f., was described by Hooker in 1886 from a Bhutanese specimen collected by Booth [36], followed by descriptions of additional species such as *S. chinensis* Allen [37], *S. hongkongensis* N. H. Xia, Y. F. Deng & K.L. Yip [38], and *Sinopora hsiwenii* Bing Liu & Y. Yang (2008, Hunan) [39]. Taxonomic debates have occurred, with some authors, including Kostermans and Rohwer, advocating for merging *Syndiclis* into *Potameia* [40,41], whereas others, such as Li X.W., Bai P.Y., and Li J., supported maintaining *Syndiclis* as a distinct genus based on diagnostic traits, including sterile stamen count and glandular positions [42,43]. In 2008, J. Li et al. reassigned *S. hongkongensis* to the newly established genus *Sinopora* based on floral merism [43]; however, due to complex floral variation and advances in morphological and molecular data, current taxonomic consensus generally treats Chinese species of *Syndiclis* and *Sinopora* as a single genus [31,44], a view embraced in this study. Currently, research on the geographic distribution of this genus remains limited, with its distribution centers and future trends still unclear. Conservation priorities are lacking, and studies on habitat suitability modeling and the identification of key environmental drivers are notably absent.

In this study, we investigate the genus *Syndiclis* (Lauraceae) to evaluate its potential distribution and key environmental constraints under current and future climate scenarios. Using the MaxEnt model integrated with ArcGIS, we combined topographic, climatic, and edaphic variables to assess the impacts of climate change on habitat suitability and species range dynamics. This approach allows us to identify climatically suitable areas, track spatiotemporal shifts in distribution, and determine dominant environmental drivers. Specifically, we aim to (1) predict suitable habitats for *Syndiclis* under present climate conditions, (2) identify major contributing variables using the Jackknife test, and (3) analyze distributional changes under future scenarios. Our findings provide a scientific foundation for conservation planning, ex situ preservation, and sustainable use of *Syndiclis*, particularly in regions vulnerable to climate change.

## 2. Results

### 2.1. Model Performance and Accuracy Assessment

**Figure 1:**
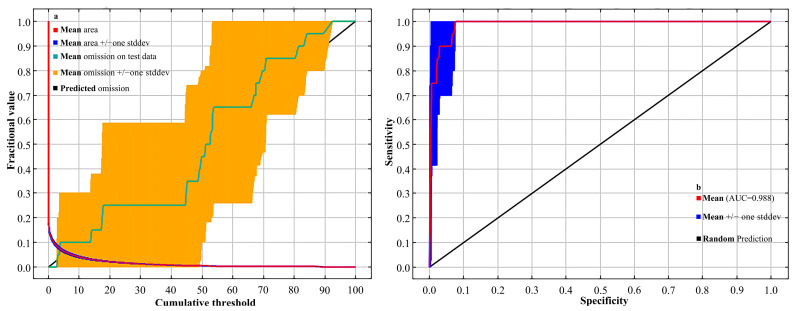
The validation of the MaxEnt model predicting *Syndiclis* distribution: (**a**) omission rate and (**b**) ROC curve.

The MaxEnt model exhibited strong performance under the default parameter settings (regularization multiplier = 1; feature classes = LQHPT), as indicated by the area under the receiver operating characteristic (ROC) curve (AUC = 0.988) (Figure 1). An AUC value approaching 1.0 reflects excellent model performance, and values above 0.9 are generally regarded as highly accurate. These results confirm that the model provides reliable predictions of the potential distribution of *Syndiclis* species and meets the accuracy standards required for ecological niche modeling in this study.

### 2.2. Dominant Environmental Variables Influencing the Distribution of Syndiclis

**Figure 2:**
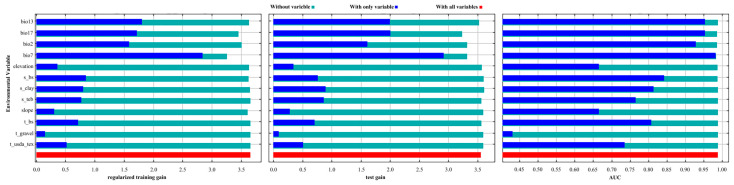
Jackknife test for environmental variables.

As shown in Table 1, two environmental variables contributed more than 10% to the model: temperature annual range (bio7, 67.00%) and precipitation of the driest quarter (bio17, 14.90%). Permutation importance analysis further confirmed the dominance of bio7 (78.50%), followed by mean diurnal range (bio2, 9.90%). As illustrated in Figure 2, four variables—bio7, bio13, bio17, and bio2—showed high contributions to model performance. Accordingly, six key environmental predictors were identified: bio2 and bio7 (temperature-related), bio13 and bio17 (precipitation-related), and s_bs and s_clay (soil-related). These six variables collectively accounted for 91.80% of the total contribution, with bio7 ranking highest, followed by bio17 and bio2. Temperature-related variables had a more substantial effect on the predicted distribution of *Syndiclis* than precipitation or soil factors. This highlights the critical role of thermal variability—especially temperature annual range (bio7)—in shaping the ecological niche of this genus.

### 2.3. Response Ranges of Dominant Environmental Variables

**Figure 3:**
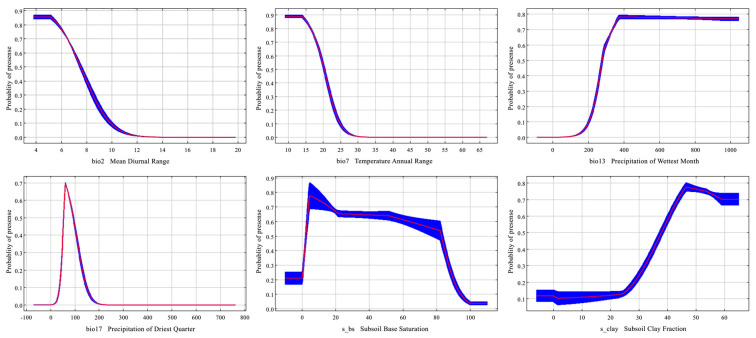
Response curves of *Syndiclis* to important climatic factors. The red lines represent the mean, while the blue represent the standard deviation for 10 replications.

Figure 3 illustrates the response curves of six dominant environmental variables influencing the potential distribution of *Syndiclis*. These include temperature-related variables (bio2 and bio7), precipitation-related variables (bio13 and bio17), and soil variables (s_bs and s_clay). For temperature, *Syndiclis* exhibited optimal conditions when the mean diurnal range (bio2) ranged between 5.12 °C and 7.52 °C, with maximum suitability (0.86) at 5.12 °C. The annual temperature range (bio7) showed a suitable interval of 13.91–20.29 °C, peaking at 0.89 at 13.91 °C. Regarding precipitation, the most suitable range for bio13 (precipitation of the wettest month) was 277.63–950.38 mm, with highest suitability (0.78) at 371.95 mm; for bio17 (precipitation of the driest quarter), suitability was highest (0.70) at 60.52 mm within a range of 53.85–93.52 mm. Soil variables also influenced habitat suitability: base saturation (s_bs) ranged from 2.34% to 82.76%, with peak suitability (0.78) at 4.76%, while optimal clay content (s_clay) was between 37.85% and 59.09%, with maximum suitability (0.78) at 46.49%. These results underscore the narrow environmental thresholds of *Syndiclis*, particularly with respect to thermal and hydrological conditions, which play a pivotal role in determining its ecological niche.

### 2.4. Current and Future Potentially Suitable Habitats for Syndiclis in China

**Figure 4:**
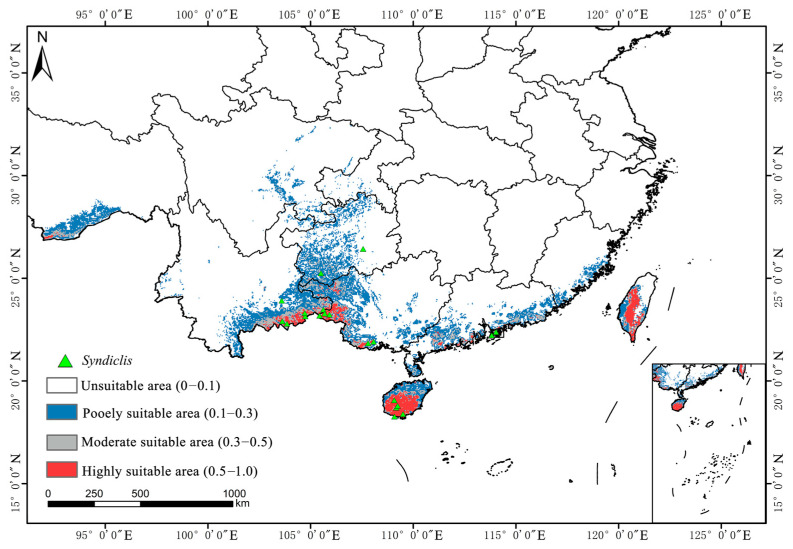
Distribution of suitable areas of *Syndiclis* under current climatic conditions.

**Figure 5:**
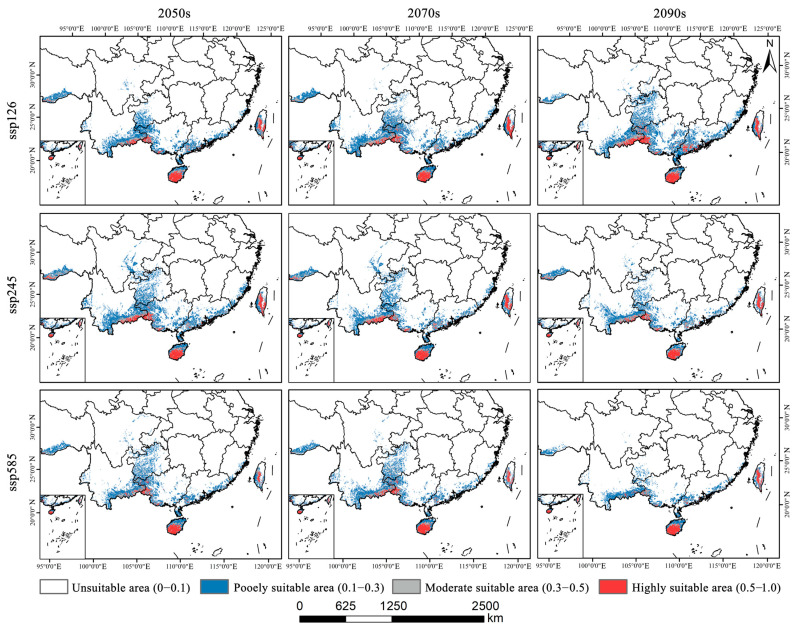
Projected distribution of suitable areas for *Syndiclis* under future climatic conditions.

**Figure 6:**
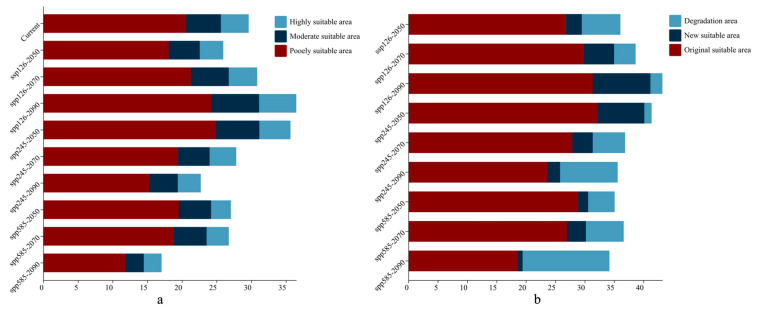
Areas (**a**) and area changes (**b**) of predicted suitable habitat in different climatic periods based on MaxEnt model.

As illustrated in Figure 4, Figure 5 and Figure 6a, the total area of potentially suitable habitat for *Syndiclis* in China under current climate conditions is approximately 29.63 × 10^4^ km^2^, including 4.02 × 10^4^ km^2^ of highly suitable habitat, 5.02 × 10^4^ km^2^ of moderately suitable habitat, and 20.60 × 10^4^ km^2^ of marginally suitable habitat. Future climate projections reveal distinct trends under different scenarios. Compared to the present, SSP126 generally predicts an expansion of suitable habitat, while SSP245 and SSP585 suggest a contraction, with the most pronounced reduction occurring under SSP585. As illustrated in Table 1, by 2050, the suitable area is projected to decrease to 25.97 × 10^4^ km^2^ (3.39 × 10^4^ km^2^ highly suitable) under SSP126, expand to 35.66 × 10^4^ km^2^ (4.49 × 10^4^ km^2^) under SSP245, and slightly decline to 27.07 × 10^4^ km^2^ (2.86 × 10^4^ km^2^) under SSP585. In 2070, SSP126 continues to show a slight expansion (30.85 × 10^4^ km^2^; 4.09 × 10^4^ km^2^ highly suitable), while SSP245 and SSP585 project declines to 27.84 × 10^4^ km^2^ (3.85 × 10^4^ km^2^) and 26.76 × 10^4^ km^2^ (3.19 × 10^4^ km^2^), respectively. By 2090, SSP126 predicts the largest expansion (36.50 × 10^4^ km^2^; 5.37 × 10^4^ km^2^), whereas SSP245 and SSP585 project significant contractions to 22.72 × 10^4^ km^2^ (3.31 × 10^4^ km^2^) and 17.06 × 10^4^ km^2^ (2.56 × 10^4^ km^2^), respectively. Currently, *Syndiclis* is mainly distributed in Southern China, particularly in Hainan, Taiwan, Southeastern Yunnan, and Southwestern Guangxi. Although the extent of suitable habitats varies across climate scenarios, these core distribution areas are expected to remain relatively stable and may serve as long-term climatic refugia.

### 2.5. Spatiotemporal Shifts in Syndiclis Distribution Under Future Climate Scenarios

**Figure 7:**
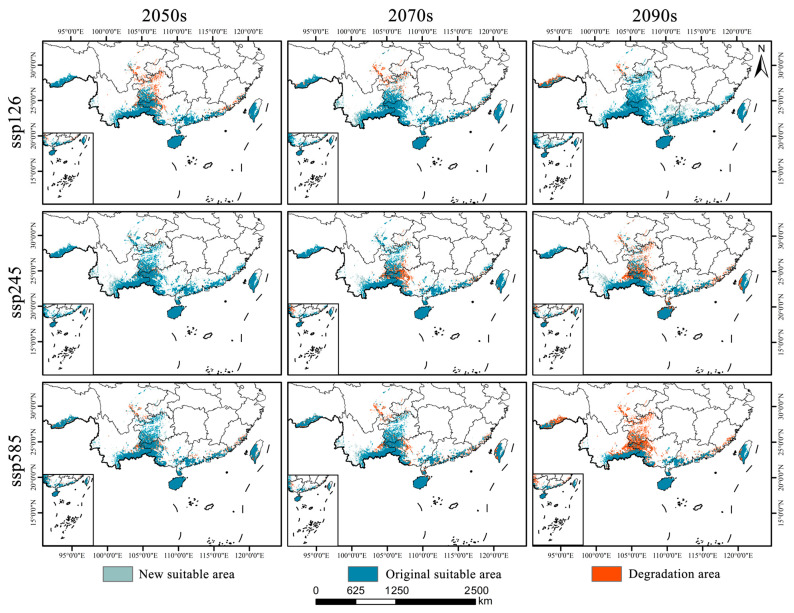
Spatial change patterns of suitable habitats of *Syndiclis* under different climate scenarios.

As shown in Figure 6b and Figure 7, *Syndiclis* is projected to undergo significant shifts in suitable habitat under future climate scenarios, with substantial variations in both magnitude and direction across different time periods and Shared Socioeconomic Pathways (SSPs). Compared to the current distribution, SSP126 shows an initial contraction followed by expansion; SSP245 exhibits the opposite trend; and SSP585 presents a continuous decline. By 2050, SSP126 predicts the greatest habitat loss (22.16%) with limited gain (9.04%), whereas SSP245 forecasts the largest expansion (26.98%) and the smallest loss (4.17%). SSP585 projects moderate loss (15.18%) and minimal gain (5.71%). By 2070, SSP126 will shift toward expansion (17.78% gain vs. 12.32% loss); SSP245 will remain relatively stable (11.74% gain vs. 18.59% loss), and SSP585 will continue to decline (21.66% loss, 10.92% gain). By 2090, expansion under SSP126 becomes more prominent (33.18% gain, 6.85% loss), while SSP245 shows net contraction (33.04% loss, 7.47% gain), and SSP585 exhibits the most severe decline, with nearly half (49.81%) of suitable habitats lost and only 2.73% newly gained. Regionally, habitat loss is primarily concentrated in Sichuan, Guizhou, and Guangxi, whereas gains are predominantly observed in Yunnan and Guangdong. Suitable areas in Hainan and Taiwan remain relatively stable, although slight variations in suitability levels are anticipated.

### 2.6. Centroid Shift Analysis

**Figure 8:**
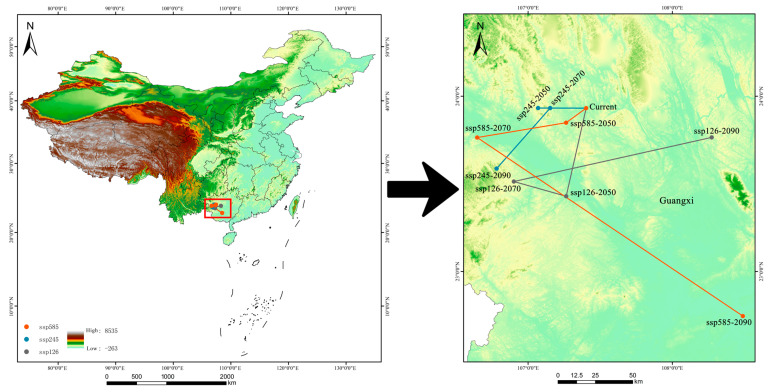
The centroid change in suitable habitats of *Syndiclis* under different climate scenarios.

As illustrated in Figure 8, the current centroid of suitable habitat for *Syndiclis* is located in Hechi City, Guangxi. Under SSP126, the centroid is expected to shift 57.62 km southwest to Baise by 2050, move 38.20 km northwest within Baise by 2070, and migrate 142.66 km northeast to Nanning by 2090. In the SSP245 scenario, the centroid is expected to shift 33.91 km westward within Hechi by 2050, followed by a slight northward displacement of 8.55 km by 2070, and a further southwestward movement of 54.02 km to Baise by 2090. Under the SSP585 scenario, the centroid is projected to shift 16.90 km southwest within Hechi by 2050, continue 63.53 km southwest to Baise by 2070, and finally shift significantly southeastward by 219.78 km to Nanning by 2090. Overall, centroid trajectories vary considerably across both scenarios and timeframes. By 2050, all scenarios exhibit moderate latitudinal displacements, generally trending toward lower latitudes and higher elevations. In 2070, the directional patterns diverge: SSP126 indicates a northwestward shift; SSP245 shows a minor northward movement, and SSP585 trends southwestward—all maintaining a tendency toward higher elevations. By 2090, shifts become more pronounced in both direction and topographic context: SSP126 projects a northeastward movement toward lower-elevation regions; SSP245 indicates a southwestward migration toward mountainous zones, and SSP585 reveals a marked southeastward shift toward lowland areas.

### 2.7. Landscape Pattern Analysis

**Figure 9:**
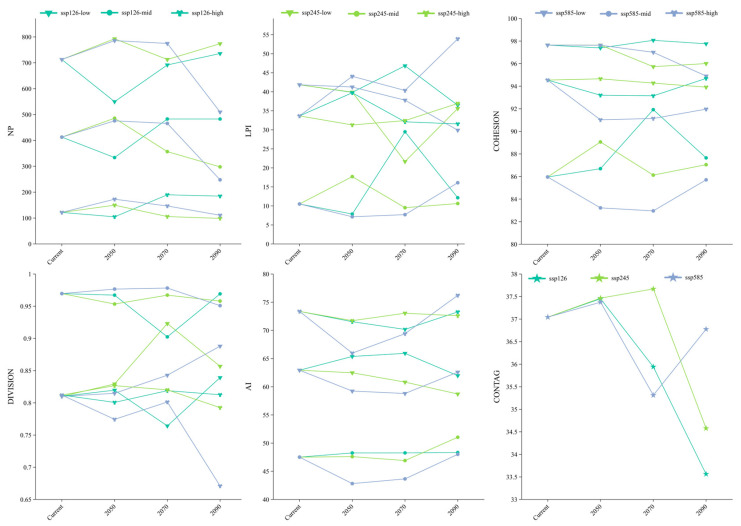
Potential suitable area landscape index change of wild *Syndiclis* species.

Figure 9 illustrates notable changes in landscape metrics of *Syndiclis* suitable habitats under future emission scenarios. The patch number (NP) in highly suitable areas remained relatively stable, ranging from 98 to 189. In contrast, medium and low suitability zones showed divergent trends. Under SSP126, NP initially declined and then increased, while under SSP585, it followed the opposite pattern. The largest patch index (LPI) generally decreased in low suitability areas. Specifically, under SSP585, LPI steadily declined. Under SSP126, it showed a dip–rise–fall pattern. SSP245 caused a sharp increase in LPI after an initial decline. Cohesion (COHESION) remained stable in low suitability zones. It declined in medium zones but increased in highly suitable areas, although these changes varied with scenarios. Division (DIVISION) was relatively unchanged overall but was highest in medium suitability zones, indicating greater fragmentation there. Highly suitable areas showed consistent aggregation, reflected by low division values. The aggregation index (AI) was stable in low suitability zones. In medium and high zones, it generally increased, except for a steady decline under SSP126. High suitability zones showed a pattern of decline followed by recovery across all scenarios. Finally, the contagion index (CONTAG) declined across all scenarios by 2090, indicating reduced spatial connectivity among suitable habitats. SSP126 and SSP245 exhibited initial increases followed by decreases in CONTAG, whereas SSP585 showed a wave-like fluctuation, suggesting distinct spatial dynamics across scenarios.

### 2.8. Gap Analysis of Conservation Coverage

**Figure 10:**
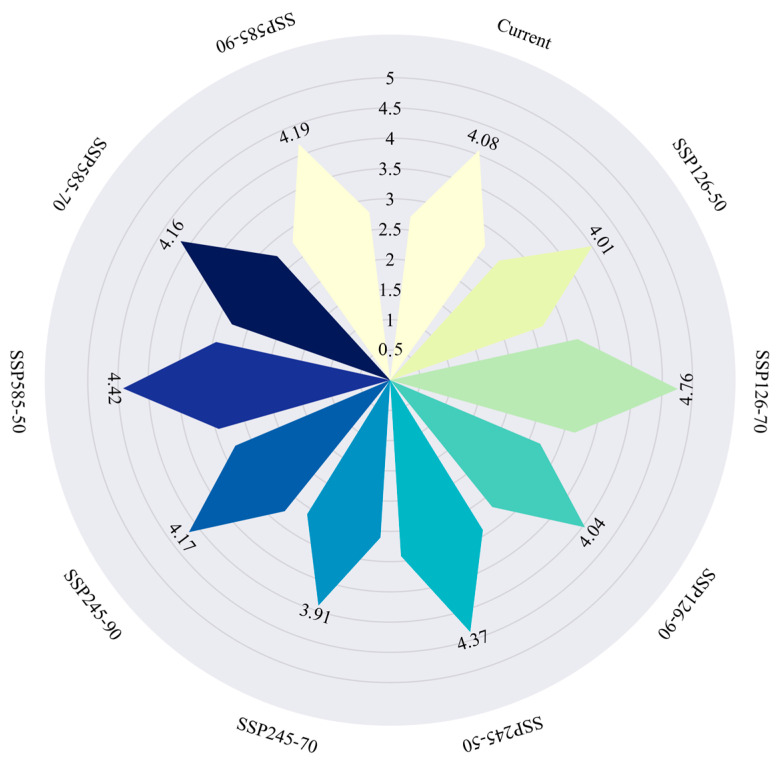
Area ratio of protected areas (the coverage area ratio of the priority protection area).

**Figure 11:**
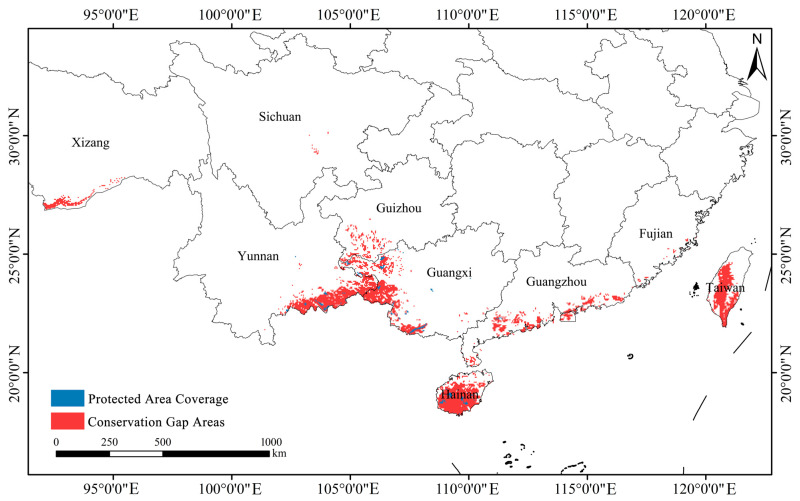
Protection gap of *Syndiclis* species.

Based on predictions of highly suitable habitat for *Syndiclis*, the total area of priority conservation areas is estimated at approximately 90,300 km^2^. However, only 3700 km^2^ (4.08%) of these areas currently fall within the boundaries of established nature reserves, indicating that 95.92% of critical habitats remain outside existing protection frameworks (Figure 10). Spatial distribution analysis (Figure 11) reveals marked regional disparities in conservation coverage. Most existing reserves are confined to parts of Yunnan, Hainan, and Guangxi, whereas large portions of high-priority habitats in Guizhou, Taiwan, and other areas of Yunnan remain beyond the formal conservation network. These findings highlight a substantial conservation shortfall and emphasize the urgent need for region-specific protection strategies.

## 3. Discussion

### 3.1. Model Accuracy Assessment and Conservation Gap Analysis

The predictive performance of the MaxEnt model is influenced by both species occurrence data and environmental variables. Its reliability depends largely on the selection of the ecological niche model, the quality and spatial coverage of occurrence records, and the type and number of environmental predictors used [45]. Ecological niche models serve as effective tools for inferring the climatic requirements of species and projecting their potential distributions across spatial and temporal gradients. Divergent emission trajectories and GCM uncertainties across SSPs propagate habitat prediction variability. MaxEnt inherently assumes species–environment equilibrium and excludes biotic interactions/dispersal constraints. We emphasize cautious interpretation and advocate for future integration of dynamic processes with field validation, using the MaxEnt model with ArcGIS to assess *Syndiclis* distribution in relation to environmental variables and future climate scenarios. Spatial filtering reduced sampling bias, while multicollinearity was addressed through correlation analysis, variable importance ranking, and jackknife testing [46]. Model performance (AUC = 0.988) was evaluated via ROC curves. The model’s performance was evaluated using the receiver operating characteristic (ROC) curve, yielding an AUC value of 0.988. This value is consistent with previous findings for related taxa, including *Camphora* [24], *Litsea* [47], *Woonyoungia septentrionalis* [48], and *Michelia odora* [49], confirming the model’s high reliability and strong predictive accuracy in capturing the distribution patterns of *Syndiclis*.

Scientifically predicting the potential distribution of rare and endangered plant species and establishing wild germplasm banks and nature reserves have become essential strategies for biodiversity conservation [50]. Currently, *Syndiclis*, as a rare and ecologically specialized genus, faces several challenges, including sparse and uneven natural distribution, insufficient scientific investigation, limited natural regeneration of wild populations, and ongoing population decline. All species within this genus are narrow-range endemics, making them particularly susceptible to climate change impacts [51,52]. Due to their strict hydrothermal requirements and weak climatic adaptability, *Syndiclis* species are likely to face further habitat reductions under future climate scenarios. Our conservation gap analysis revealed that only 4.08% of priority conservation areas are currently covered by existing nature reserves, highlighting a significant shortfall in protection. Therefore, identifying and designating priority conservation units is critical for formulating effective conservation strategies in the face of climate change. In addition, we recommend strengthening forest management and supervision, raising public awareness about plant conservation, promoting the importance of plant protection in the context of ecological civilization, and implementing artificial afforestation or assisted restoration when necessary.

### 3.2. The Dominant Factors That Restrict the Distribution of Syndiclis Habitat

The interaction between environmental factors and species distribution is a central theme in ecology, biogeography, and conservation management of rare and endangered plants [53]. Species’ geographical distribution is regulated by multiple environmental variables and reflects the outcome of long-term species–environment interactions. Climate change, as a major determinant of global vegetation patterns, directly influences species distribution, migration, and survival [54]. In the present study, six variables, bio2, bio7, bio13, bio17, S_bs, and S_clay, were identified as key factors shaping the potential distribution of *Syndiclis*, among which bio7 (temperature annual range) contributed up to 67.00%, serving as the dominant determinant. Hydrothermal conditions are known to be major bioclimatic drivers for woody plants, but their specific influence often varies across species. Temperature plays a pivotal role in plant growth, development, and biogeography [55]. For *Syndiclis*, temperature was found to be the most significant limiting factor, followed by precipitation, while soil variables had relatively minor effects. This is consistent with findings in other Lauraceae species. For instance, Li Hui et al. [24] reported that temperature-related factors primarily constrained the distribution of rare and endangered *Camphora* species. Zhou Run et al. [56] further noted that while most *Camphora* species are influenced by hydrothermal conditions, some are mainly governed by temperature. *Similarly*, *Cassytha* L. distribution was predominantly shaped by temperature, which exerted a stronger effect than precipitation [57]. In contrast, studies on *Litsea* Lam. indicated precipitation as the dominant factor, with temperature being secondary [47], slightly differing from our results, though hydrothermal variables still played the central role. Other studies have confirmed temperature as the primary determinant in species such as *Salix tetrasperma* Roxb. [58], alpine *Quercus* sect. Heterobalanus in the *Hengduan Mountains* [59], evergreen Sclerocarpus oaks [60], and endangered *Horsfieldia hainanensis* Merr. [22]. *Woonyoungia septentrionalis*, a rare species, was mainly influenced by combined hydrothermal conditions [48], aligning with our findings. Overall, species exhibit distinct responses to environmental variables, and the dominant factors governing their potential habitats are often species-specific [61]. These differences underscore the importance of tailored strategies in conservation planning and habitat suitability modeling.

Hydrothermal conditions significantly influence the germination, growth, and spatial distribution of seedlings in rare and endangered plant species [62]. This is further corroborated in *Syndiclis*, a narrowly distributed genus highly sensitive to environmental changes. Its flowering phenology is notably affected by temperature and precipitation [63], and the link between phenology and hydrothermal conditions has been similarly reported in other members of the Lauraceae [64]. *Syndiclis* exhibits unique phenological traits, such as two flowering periods and one fruiting phase per year, short flowering durations, and extremely small flowers. The first flowering peak typically occurs between May and June and is fertile, during which temperature is significantly correlated with phenological development. Pollination success, a key determinant of reproductive output, is jointly influenced by flowering temperature, flower number, and anther orientation. In some species, upward-facing anthers lead to increased pollen exposure to rainfall, reducing pollen viability and further emphasizing the dominant regulatory role of temperature [39]. Additionally, *Syndiclis* predominantly inhabits limestone mountainous regions and mid- to high-elevation cloud forests—habitats characterized by thin soil layers, high rock exposure, and microclimatic variability. In these environments, soil temperature is strongly influenced by surface rocks, and thick seed coats hinder natural germination. Although winter humidity and spring rainfall can facilitate seed coat decomposition, temperature remains the principal driver of seed germination [30]. The interception of rainfall by litter and water storage in soil layers further attenuates the direct impact of precipitation. As a tropical-origin family, Lauraceae members generally require higher temperatures. Decreasing temperatures associated with higher latitudes and elevations are known to reduce species richness within this family [24], consistent with the restricted distribution patterns of *Syndiclis* observed in this study.

The declining contagion index (CONTAG) across future scenarios indicates reduced habitat connectivity, potentially impeding pollen/seed dispersal and restricting gene flow in *Syndiclis*. This fragmentation may drive smaller, isolated populations with increased inbreeding depression and diminished genetic diversity, ultimately eroding adaptive capacity. Concurrently, persistently high division index (DIVISION) in medium-suitability zones suggests stable fragmentation of critical dispersal corridors. Given *Syndiclis*’ narrow habitat specificity and limited dispersal, such landscape disintegration exacerbates climate vulnerability by reducing effective population connectivity. These findings underscore the imperative to integrate spatial metrics into conservation frameworks for fragmented-endemic taxa.

### 3.3. Current Distribution Prediction and Distribution Center of Syndiclis

This study reveals the potential spatial pattern changes and distribution centroid shifts of *Syndiclis* under different scenarios in the present and future by modeling and analyzing the species distribution data and climate data with the help of software such as MaxEnt and GIS, which is of great significance for the conservation of species and ecosystems. The results show that *Syndiclis* is mainly distributed in 103°41′–114°08′ E, 18°25′–26°26′ N, covering Hainan, Taiwan, Southwestern Guangxi, Southeastern Yunnan, Southeastern Tibet, Southeastern Guizhou, and Southeastern Sichuan, consistent with actual records. Highly suitable areas in Yunnan and Guangxi are concentrated in Xichou County, Jingxi City, and Baise City, demonstrating distributional stability. This corroborates the hypothesized origin in Southwestern China (particularly Southeastern Yunnan) [35], while we propose Hainan and Taiwan as “modern refuges” due to their persistent habitat suitability. Stable distributions in limestone mountains and mid-elevation cloud forests provide suitable microclimates and vertical migration space. However, future warming and drought intensification will reduce high-suitability habitats, forcing migration toward low-elevation/latitude humid zones. This intensifies habitat fragmentation, blocking migration routes and increasing extinction risk [65], aligning with the narrow thermal adaptation of tropical taxa [66]. *Syndiclis* exhibits significant range dynamics and centroid shifts: by 2050, contraction under SSP126/SSP585 but expansion under SSP245 are expected, with southwest centroid shifts; by 2070, only SSP126 will show expansion with directional centroid shifts; by 2090, intensified “southwest–southeast” shifts and atypical elevation changes are expected [67]. This study concluded that the reduced habitat adaptability in the west, the degradation of the northern boundary suitable area, and the complexity of topography and climate were the main causes of the southward distribution of *Syndiclis* and that the trend was driven by geographic factors rather than physiological adaptations of the species [24], and it was consistent with the results of some rare camphor tree studies, which further verified the scientific validity of this study.

Landscape index analysis further showed that with climate change, the area of suitable habitat is shrinking and habitat fragmentation is increasing. This is mainly affected by temperature factors, indicating that warmer temperatures do not universally promote habitat dispersal of plants [68]. Therefore, environmental factors such as temperature should be prioritized when introducing and domesticating species, while the distribution of species’ fitness is also limited by anthropogenic factors and other abiotic factors that affect the growth and survival of species [69]. Therefore, we should pay special attention to the changes in suitable habitats at low latitudes and low altitudes in the future study of the fitness changes in *Syndiclis* due to climate warming. In particular, we should incorporate the effects of anthropogenic factors, the species’ own characteristics, and community ecosystems into the study of the fitness distribution of the species, which can be further verified by combining with molecular studies and fossilized plants to improve the accuracy and scientificity of the simulation and prediction.

## 4. Materials and Methods

### 4.1. Data and Materials

#### 4.1.1. Species Occurrence Data

To collect occurrence data for *Syndiclis* species, two complementary approaches were employed. First, 12 presence records were obtained from field investigations conducted in natural habitats across Southern China. Second, additional occurrence data were compiled from multiple secondary sources, including the Chinese Virtual Herbarium (CVH; https://www.cvh.ac.cn/, accessed on 5 January 2025), the Global Biodiversity Information Facility (GBIF; https://www.gbif.org/, accessed on 10 January 2025), the published literature, and floristic monographs. This process yielded a total of 95 raw occurrence records within China. To improve data quality and reduce spatial autocorrelation and potential model overfitting, duplicated and erroneous records were removed. Furthermore, to account for sampling bias, only one occurrence point was retained within each 5 km × 5 km grid cell. This spatial filtering was performed using the SDMToolbox v2.5 implemented in ArcGIS 10.8. After filtering, a total of 20 valid occurrence points were retained for modeling. Each record contained species name, longitude, and latitude, and the final dataset was exported in CSV format for subsequent ecological niche modeling.

#### 4.1.2. Environmental Data Sources and Variable Selection

Environmental variables used in this study encompassed climate, topography, soil, and ultraviolet (UV) radiation factors, totaling 60 variables. Nineteen bioclimatic variables (bio1-bio19) were obtained from WorldClim version 2.1 (https://www.worldclim.org/, accessed on 1 March 2025), representing current conditions as well as future projections for the 2050s (2041–2060), 2070s (2061–2080), and 2090s (2081–2100). Future climate data were based on the Beijing Climate Center Climate System Model version 2.1 (BCC-CSM2-MR), a coupled global climate model widely used for regional climate projections, with a spatial resolution of 2.5 arc-minutes. Three Shared Socioeconomic Pathway (SSP) scenarios, SSP126, SSP245, and SSP585, were selected to capture a range of potential future climate conditions. Topographic variables, including slope, aspect, and elevation, were derived from a digital elevation model (DEM) provided by the Geospatial Data Cloud (http://www.gscloud.cn/, accessed on 5 March 2025). These variables were computed using spatial analysis tools in ArcGIS 10.8. Soil data comprising 32 variables were sourced from the Harmonized World Soil Database (HWSD, https://www.fao.org/soils-portal/data-hub/soil-maps-and-databases/harmonized-world-soil-database-v20/en/, accessed on 10 March 2025). Additionally, six UV radiation variables were acquired from the Global UV Radiation Database (https://www.ufz.de/gluv/, accessed on 15 March 2025). The base map used for modeling was obtained from the Standard Map Service System of the Ministry of Natural Resources of China (http://bzdt.ch.mnr.gov.cn/, accessed on 20 March 2025). To ensure consistency and model stability, all environmental raster layers were converted to ASCII format, reprojected to a common geographic coordinate system (GCS-WGS1984), and resampled to a uniform spatial resolution of 2.5 arc-minutes using ArcGIS 10.8. Given the potential for multicollinearity among predictor variables, we implemented a multi-step variable selection process to optimize model performance and reduce overfitting. First, a preliminary MaxEnt run was conducted to assess the importance and contribution of each variable via Jackknife tests. Next, environmental values were extracted at species occurrence points using ArcGIS extraction tools, and pairwise Pearson correlation coefficients were calculated to evaluate inter-variable correlations. Variables with an absolute correlation coefficient |r| > 0.80 were considered highly correlated, and among these, those with negligible contribution to the model prediction (contribution rate = 0) were excluded. The final set of variables was selected based on ecological relevance and statistical criteria following established protocols [18,19], which has been shown to improve simulation accuracy [20]. Ultimately, the optimal variable set included four climatic variables (bio2: Mean Diurnal Range, bio7: Temperature Annual Range, bio13: Precipitation of Wettest Month, bio17: Precipitation of Driest Quarter), six soil variables (t_usda_tex: USDA soil texture, t_bs: base saturation, t_gravel: gravel content, s_bs: soil base saturation, s_clay: clay content, s_teb: total exchangeable bases), and two topographic variables (slope, elevation) (Table 2).

### 4.2. Species Distribution Modeling and Model Accuracy Assessment

In this study, the accuracy of the MaxEnt model was evaluated using the area under the receiver operating characteristic curve (AUC-ROC) [70]. The AUC value is minimally affected by sample size and threshold selection, making it a robust metric for assessing the predictive performance of MaxEnt models. As shown in Figure 1, the red curve represents the training dataset’s ROC analysis, with AUC values ranging from 0.5 to 1.0, where values closer to 1.0 indicate higher model accuracy [71]. Based on the criteria established by Kumar et al. [72], AUC values are interpreted as follows: 0.5–0.6 indicates no predictive ability; 0.6–0.7 indicates low accuracy; 0.7–0.8 represents moderate accuracy; 0.8–0.9 denotes high accuracy; and 0.9–1.0 indicates excellent predictive performance [73,74]. A total of 20 occurrence records and 12 environmental variables were input into the MaxEnt model. The dataset was randomly split, with 75% used for model training and 25% reserved for testing, to ensure robust model validation. Response curves for individual environmental variables were generated to evaluate the species’ habitat suitability. A predicted presence probability (*p*) of ≤0.5 was interpreted as low habitat suitability, whereas *p* > 0.5 indicated high suitability [75]. Variable importance and contribution were assessed using the Jackknife test, with default MaxEnt parameters maintained. Ten replicate runs were performed to enhance result reliability, culminating in the production of species distribution prediction maps.

### 4.3. Classification of Habitat Suitability Levels, Spatial Dynamics, and Environmental Variable Importance Assessment

The output of the MaxEnt model was visualized using ArcGIS 10.2. Habitat suitability was classified into four categories using the Reclassify tool combined with the Natural Breaks (Jenks) method. Threshold values were manually set to define the following suitability classes: unsuitable area (0–0.1), poorly suitable area (0.1–0.3), moderately suitable area (0.3–0.5), and highly suitable area (0.5–1.0). The suitable habitat area of *Syndiclis* was quantified for different time periods, and its distributional changes under various future climate scenarios were compared. In this study, regions with a habitat suitability index ≥ 0.1 were considered suitable habitats, whereas those with a suitability index < 0.1 were classified as unsuitable. Presence/absence (binary) matrices were constructed for *Syndiclis* suitability distributions under future climate scenarios, where “1” indicated presence (suitable habitat), and “0” indicated absence (unsuitable habitat). Based on these matrices, changes in habitat suitability were categorized into four types: new suitable areas (areas becoming suitable), degradation areas (areas losing suitability), original suitable areas (areas consistently suitable), and unsuitable areas (consistently unsuitable).

### 4.4. Habitat Landscape Pattern Analysis

Landscape pattern metrics serve as key indicators to characterize spatial heterogeneity and structural attributes of habitats, enabling systematic analysis of habitat fragmentation, patch connectivity, and spatial configuration [76]. In this study, Fragstats 4.2 software was employed to calculate six landscape metrics at both the class and landscape levels: Patch Number (NP), Largest Patch Index (LPI), Aggregation Index (AI), Landscape Division Index (DIVISION), Patch Cohesion Index (COHESION), and Contagion Index (CONTAG). These metrics were used to analyze the fragmentation degree, aggregation status, and heterogeneity of *Syndiclis*’ potential suitable habitats under different climate scenarios, thereby elucidating the dynamic changes in landscape patterns.

### 4.5. Species Distribution Centroid Migration Analysis

The direction and distance of species habitat suitability shifts can be inferred from changes in the geographic centroid of suitable habitats [77]. Using ArcGIS and the SDM_Toolbox, the geometric centroids of current and future suitable habitat distributions (binary maps) were identified. The latitude and longitude coordinates of these centroids were extracted, and centroid shifts and migration distances of *Syndiclis* under various future climate scenarios were calculated [78].

### 4.6. Conservation Gap Analysis

Potential highly suitable habitat areas of *Syndiclis* were designated as priority conservation zones. Using ArcGIS, highly suitable areas were extracted for each temporal climate scenario. The spatial boundaries of existing global nature reserves were applied as masks via the Extract by Mask tool to identify overlaps between *Syndiclis* priority habitats and protected areas. Changes in the extent of protected suitable habitats were quantified across different time periods to assess the impact of future climate change on the spatial distribution and coverage of *Syndiclis*’ potential conservation areas.

## 5. Conclusions

This study integrates species distribution modeling (MaxEnt), landscape pattern analysis, and conservation gap assessment to evaluate the climate-driven distributional shifts and conservation risks of the rare laurel genus *Syndiclis* in China. The species’ distribution is strongly influenced by thermal factors, particularly the temperature annual range (Bio7), with a narrow thermal niche (13.91–20.29 °C; <6.4 °C amplitude), indicating high climate sensitivity. Compared to other Lauraceae, *Syndiclis* exhibits stricter thermal tolerance, increasing its vulnerability to warming. Projections show divergent outcomes: potential range expansion under SSP126 and significant contraction (up to −42.4%) under SSP585. Centroid shifts toward lower latitudes and mid-to-high elevations suggest that karst terrains may serve as climate refugia. However, habitat fragmentation intensifies under high-emission scenarios, leading to increased isolation, reduced connectivity, and potential gene flow disruption. Conservation gap analysis reveals that only 4.08% of high-suitability areas—mainly in Southeastern Yunnan, Southwestern Guangxi, and Hainan—are currently protected, underscoring urgent conservation needs. To address this, a three-tiered strategy is proposed: (1) Core Climate Refugia—stable, high-suitability zones needing strict protection and microclimate monitoring; (2) Buffer Restoration Zones—adjacent habitats to be restored using Lauraceae species and substrate improvements; and (3) Climate-Adaptive Corridors—linking shifting habitats with ex situ stations to maintain gene flow and adaptive potential. *Syndiclis* provides a model for understanding climatic limits in edge-tropical taxa, emphasizing the need for integrated, forward-looking conservation strategies supported by ecological and paleoenvironmental data.

## Figures and Tables

**Table 1:** Projected changes in suitable habitats of *Syndiclis* under future climate scenarios.

Year	Scenario	Total Suitable Area (×10^4^ km^2^)	Highly Suitable Area (×10^4^ km^2^)	Change Trend
2050	SSP126	25.97	3.39	Decrease
	SSP245	35.66	4.49	Increase
	SSP585	27.07	2.86	Slight decrease
2070	SSP126	30.85	4.09	Slight increase
	SSP245	27.84	3.85	Decrease
	SSP585	26.76	3.19	Decrease
2090	SSP126	36.5	5.37	Major increase
	SSP245	22.72	3.31	Significant decrease
	SSP585	17.06	2.56	Drastic decrease

**Table 2:** Parameters of Environmental Variables.

Code	Environmental Variable	Contribution Rate/%	Permutation Importance/%
bio7	Temperature Annual Range	67.00	78.50
bio17	Precipitation of Driest Quarter	14.90	3.40
bio2	Mean Diurnal Range	3.60	9.90
slope	slope	2.50	2.60
s_bs	Subsoil Base Saturation	2.60	1.40
t_usda_tex	Topsoil USDA Texture Classification	2.50	0.10
s_clay	Subsoil Clay Fraction	2.10	0.40
elevation	Elevation	1.80	3.10
bio13	Precipitation of Wettest Month	1.60	0.50
t_bs	Topsoil Base Saturation	0.30	0.10
t_gravel	Topsoil Gravel Content	0.10	0
s_teb	Subsoil TEB	0.10	0

## Data Availability

Data are contained within this article.
